# Boosting visible-light photocatalysis with MWCNT-modified TiO_2_/SiO_2_/g-C_3_N_4_: efficient tetracycline removal in pure and hard water

**DOI:** 10.1038/s41598-026-39505-4

**Published:** 2026-02-09

**Authors:** Samira Mohammaddarvish, Amir Ali Masoudi, Zahra Sadat Hosseini

**Affiliations:** 1 Department of Condensed Matter Physics, Faculty of Physics, Alzahra University, Tehran, Iran; 2 Photonics and Quantum Technologies Research School, Nuclear Science and Technology Research Institute (NSTRI), Tehran, Iran

**Keywords:** TiO_2_/SiO_2_/g-C_3_N_4_/MWCNT composite, Photocatalyst, Visible light, Methylene blue, Tetracycline, Chemistry, Environmental sciences, Materials science, Nanoscience and technology

## Abstract

In this study, we present a facile synthesis of a TiO_2_/SiO_2_/g-C_3_N_4_/MWCNT composite with dual functionality as both a photocatalyst and adsorbent for the effective elimination of pharmaceutical and dye pollutants from water. The composite was comprehensively characterized using FESEM, EDX, XRD, BET, FTIR, PL, and UV–Vis spectroscopy to elucidate its morphology, crystalline structure, and optical properties. The results revealed a hierarchical flower-like sphere/sheet architecture with an average crystallite size of about 21.3 nm. Multi-walled carbon nanotubes (MWCNTs) acted as visible-light photosensitizers, enhancing the separation efficiency of photogenerated electron–hole pairs. The influence of MWCNT content on photocatalytic activity was systematically investigated. The TiO_2_/SiO_2_/g-C_3_N_4_/MWCNT composite with 11 wt% CNT demonstrated superior visible-light degradation performance, achieving 92% efficiency for a 20 mg/L methylene blue (MB) aqueous solution significantly outperforming the MWCNT-free counterpart. Furthermore, the presence of MWCNTs remarkably improved tetracycline (TC) removal in CaCO_3_-rich water (20 mg/L TC with 300 mg/L CaCO_3_). The high photocatalytic activity is attributed to the formation of abundant local junctions among g-C_3_N_4_, TiO_2_/SiO_2_, and MWCNTs, which facilitate efficient charge separation and migration through a direct Z-scheme mechanism under visible light illumination.

## Introduction

The rapid growth of the global population and industrialization in recent decades has had detrimental impacts on the environment, particularly on water quality. Increased human activity has introduced substantial amounts of organic, inorganic, and microbial pollutants into natural water bodies^1,2^. Coastal ecosystems are especially vulnerable to contamination, and the widespread use of antibiotics in medical and industrial applications has led to serious water pollution concerns. Among these, TC, a widely used antibiotic, poses a significant environmental and public health threat. TC contamination contributes to the proliferation of antibiotic-resistant bacteria and adversely affects aquatic life. Moreover, it is persistent in the environment, leading to long-term ecological risks and potential harm to human health. The main sources of TC pollution include improper disposal of pharmaceuticals by manufacturers, excretion from humans and livestock, and runoff from animal husbandry and aquaculture^3–5^. Due to its poor biodegradability and resistance to conventional treatment methods, effective removal of TC from water sources remains an urgent challenge. Therefore, developing advanced remediation strategies for TC degradation is of critical importance^1–6^. Among all water purification methods such as adsorption, nano-filtration, and sedimentation, the photocatalytic degradation is an environmentally friendly method which can eliminate pharmaceutical pollutants. By absorbing light, the photocatalyst generates electron–hole (e^−^/h^+^) pairs that lead to the formation of hydroxyl radicals (^·^OH) and superoxide radicals (^·^O_2_^**−**^). These reactive species degrade a wide range of organic and inorganic pollutants, converting them into less harmful or non-toxic byproducts^7,8^.

Since visible light constitutes a significant portion of the solar spectrum, extensive research efforts have been directed toward the use of visible-light driven photocatalysts for refinement of the wastewater^9–12^. Titanium dioxide (TiO_2_) is widely recognized as a leading photocatalyst due to tunable electronic and optical properties, chemical stability, and non-toxicity which its composition with silicon dioxide (SiO_2_) enhances the photocatalytic performance^13,14^. TiO_2_/SiO_2_ photocatalyst needs to be further improved due to photoactivity in the UV region and high recombination of electron-hole pairs. To overcome these limitations, various approaches have been proposed including combination of TiO_2_ with narrow band gap catalysts^11–14^. Graphitic carbon nitride (g-C_3_N_4_) as an organic semiconductor has attracted great attention in the photocatalytic application due to non-toxic nature and robust stability under a wide pH range conditions, and photoactivity in the visible region (E_g_$\:\sim$2.7 eV). Layered structure of g-C_3_N_4_ is another desirable feature which makes its composition with other materials feasible. However, high recombination rate of photogenerated charge carriers limits the photocatalytic efficiency which can be improved through coupling with other materials^13,14^. The capability of this ternary composite in photodegradation of organic and dye pollutants has been investigated previously^15^. Compared to traditional type-II heterojunctions, heterojunctions that the Z-scheme mechanism dominates their photocatalytic process possess higher efficiency in the photodegradation of pollutants. Regarding the edge potential positions, g-C_3_N_4_ could be an excellent candidate to form the Z-scheme heterojunction with TiO_2_/SiO_2_^10^. Although the TiO_2_/SiO_2_/g-C_3_N_4_ ternary structure offers promising characteristics, it still faces limitations in charge carrier mobility, which serves as a critical factor in driving the photocatalytic reaction. For enhancing its photocatalytic activity, integration with carbonic materials such as MWCNTs could be effective. MWCNTs, with their one-dimensional (1D) tubular architecture, honeycomb-like graphene layers, high surface area, and excellent electrical conductivity, are well suited for pollutant adsorption and electron transfer. Incorporating MWCNTs into the composite facilitates electron injection and significantly inhibits the recombination of photoinduced electron–hole pairs^12,16^. For instance, researchers synthesized a TiO_2_/g-C_3_N_4_/CNT composite via a hydrothermal method to promote charge separation and reduce recombination. Their TiO_2_/g-C_3_N_4_/CNT nanostructures achieved a 98% degradation efficiency of MB under visible light irradiation within 90 min, demonstrating the efficacy of carbon nanotube integration^12^.

The presence of ions in water can hinder interactions between the surface of photocatalyst and target pollutants. In hard water, calcium carbonate (CaCO_3_) tends to deposit on the photocatalyst surface, reducing both pollutant adsorption and light absorption. Enhanced adsorption of pollutants prior to irradiation has been shown to significantly improve photodegradation efficiency^17,18^. Recent studies have emphasized the importance of combining adsorption and photocatalysis, particularly in salt-containing water systems, where the constraints of absorption such as active site saturation can be overcome by synergistic effects^13^. With growing interest in the practical deployment of photocatalysts in the real-world conditions like seawater, an increasing number of investigations have been focused on the impact of salt ions on the photocatalytic activity. For instance, Yu et al. (2020) synthesized a TiO_2_/SiO_2_/g-C_3_N_4_ composite via hydrothermal treatment, achieving 60% pollutant adsorption and 96% removal of berberine hydrochloride (10 mg/L) under visible light (300 W) in seawater within 60 min^13^. In another study, a Yb-TiO_2_–rGO nanocomposite exhibited stable phenol degradation efficiency in both pure and saline water, attributed to strong adsorption capacity (60%) mitigating the negative impact of salt ions^18^. Despite some reports in this fields, photocatalytic removal of pollutants in ion-rich water remains a significant challenge due to the interference of salt ions, such as Ca^2+^ and HCO_3_^−^, with photodegradation pathways.

In this research, a TiO_2_/SiO_2_/g-C_3_N_4_/MWCNT composite was successfully synthesized using a straightforward hydrothermal approach. Its photocatalytic efficiency in degrading pollutants was systematically evaluated in both hard water and deionized water environments for the first time. Before photocatalysis, the composite exhibited high dark adsorption capacity (~ 80%) due to its large specific surface area of 215.85 m^2^/g substantially higher than many previously reported CNT-based materials. For instance, Sharma et al. reported a TiO_2_/CNT nanocomposite with a BET surface area of 49.96 m²/g, and Shaban et al. synthesized a TiO_2_ nanoribbon/CNT composite exhibiting a BET surface area of about 102.75 m^2^/g^19,20^. This elevated adsorption enhances pollutant accumulation on the catalyst surface prior to irradiation, improving subsequent photodegradation. The photocatalytic degradation of TC was evaluated in both deionized water and CaCO_3_-rich media and the obtained results were remarkable. This comparative study elucidates the role of charge separation efficiency in mitigating ion-induced deactivation and highlights the potential of the synthesized composite for real-world water treatment applications.

## Experimental

### Synthesis of TiO_2_/SiO_2_/g-C_3_N_4_/MWCNT composite

To synthesize the TiO_2_/SiO_2_/g-C_3_N_4_ composite with a flower-like sphere/sheet morphology, 0.075 g of urea was initially dissolved in a mixture of 1.5 mL methanol and acetic acid under continuous stirring. Subsequently, 0.08 g of polyethylene glycol 4000 (PEG) was added to the solution and stirred for 15 min to ensure homogeneity. Following this, 0.14 mL of tetraethyl orthosilicate and 0.5 mL of titanium (IV) n-butoxide were added to the mixture and stirred for an additional 30 min. The resulting solution was then heated in an oven at 40 °C for 24 h to facilitate gel formation. The obtained gel was further combined with 4.3 g of urea, 12.5 mL of deionized water, and 3 g of melamine, followed by stirring for 30 min to form a uniform suspension. This suspension was transferred into a Teflon-lined stainless steel autoclave and was treated via hydrothermal process at 170 °C for 24 h. The final product was dried at 60 °C for 18 h and subsequently calcined at 520 °C in ambient air for 4 h to achieve the desired composite structure.

Then, MWCNT with different amounts of 0 wt%, 6 wt%, 11 wt%, 33 wt% and 55 wt% marked as C1, C2, C3, C4, and C5, respectively, was dissolved into 10 mL ethanol and treated by ultrasonic for 30 min. 0.01 g of TiO_2_/SiO_2_/g-C_3_N_4_ composite was added with stirring for 30 min. The mixed solution was dried for 10 h at 80 °C to obtain TiO_2_/SiO_2_/g-C_3_N_4_/MWCNT final composite.

### Photodegradation of MB

To assess the influence of MWCNT incorporation on photocatalytic degradation, 25 mg of the synthesized photocatalyst was uniformly dispersed in 50 mL of MB (20 mg/L). The suspension was maintained in the dark for 60 min to establish adsorption–desorption equilibrium. At specific time intervals, 2 mL aliquots were extracted from the reaction mixture and centrifuged at 3000 rpm for 5 min to remove the photocatalyst. The concentration of residual MB was subsequently quantified using a UV–Vis spectrophotometer at a wavelength of 665 nm. Photocatalytic reactions were carried out under visible light using an LED array (λ ≈ 400–700 nm), delivering a total irradiance of 17.2 W/m^2^ at the location of the sample (7 cm). The irradiance value was measured using a commercial solar power meter (TES-1333R) which was calibrated before measurement.

### Photocatalytic TC degradation

To simulate hard water conditions, artificial water was prepared by dissolving CaCO_3_ in deionized water to a final concentration of 300 mg/L. A 50 mL TC solution (20 mg/L) was prepared using this artificial water. Subsequently, 25 mg of the synthesized photocatalytic powder was added to the solution. The suspension was stirred in the dark for one hour to achieve adsorption–desorption equilibrium. At predetermined time intervals during photocatalytic testing, a 2 mL sample was taken from the reaction mixture and centrifuged at 3000 rpm for 5 min to separate the suspended photocatalyst particles. The residual concentration of TC was then analyzed using a UV–Vis spectrophotometer at a detection wavelength of 365 nm.

### Characterization

The synthesized powders were comprehensively characterized using multiple analytical techniques. Morphological features and elemental composition were examined via a MIRA3 TESCAN Field Emission Scanning Electron Microscope (FESEM) integrated with Energy-Dispersive X-ray Spectroscopy (EDX) for elemental quantification. Crystallographic structure was identified using X-ray Diffraction (XRD, Bruker D8 Advance), providing insights into phase purity and lattice parameters. Optical properties and photocatalytic behavior were evaluated through Diffuse Reflectance Spectroscopy (DRS, Avantes Avaspec-2048) and UV–Visible spectrophotometry (Ocean Optics HR4000), enabling band gap estimation and light absorption analysis. Chemical bonding and functional groups were investigated using Fourier-Transform Infrared Spectroscopy (FTIR, RAYLEIGH WQF-510 A). Additionally, the specific surface area and pore size distribution were measured using the Brunauer–Emmett–Teller (BET) method by a Micromeritics TriStar II Plus (USA) instrument, offering valuable data on textural properties relevant to catalytic performance. The photoluminescence (PL) spectra were recorded on Cary Eclipsed Spectrophotometer (Agilent G980A, USA). The degradation of TC was analyzed using LC–MS (UPLC platin blue knauer-LCT premier water). Mott–Schottky plots, electrochemical impedance spectroscopy, and transient photocurrent measurements were performed with an electrochemical workstation (Potentiostat/Galvanostat, IVIUM Vertex).

## Result and discussion

### Morphology and structure of composites

**Fig. 1:**
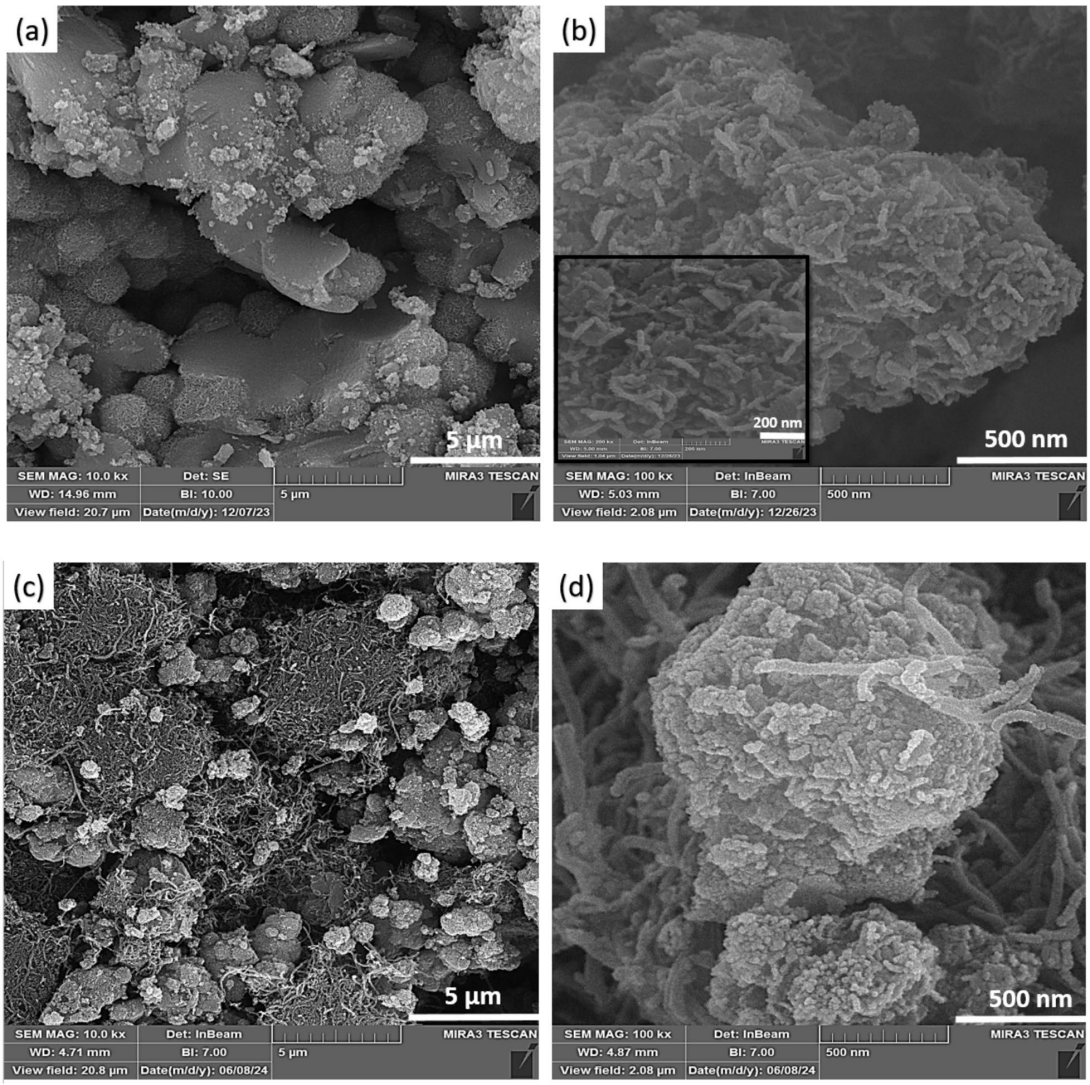
The FESEM images of (**a**,**b**) TiO_2_/ SiO_2_/g-C_3_N_4_ and (**c**,**d**) TiO_2_/ SiO_2_/g-C_3_N_4_/MWCNT composites.

The FESEM images of TiO_2_/ SiO_2_/g-C_3_N_4_ and TiO_2_/ SiO_2_/g-C_3_N_4_/MWCNT composites with 11 wt% MWCNT are illustrated in Fig. 1. The outer diameter and length of MWCNT were determined to be 20–30 nm and 10–30 μm, respectively. The FESEM images in Fig. 1a,b indicate flower-like sphere structure of TiO_2_/ SiO_2_ hybrid and sheet structure of g-C_3_N_4_ in the TiO_2_/ SiO_2_/g-C_3_N_4_ composite. Based on Fig. 1c,d, the morphology of the TiO_2_/ SiO_2_/g-C_3_N_4_/MWCNT ternary composite has been composed of the TiO_2_/SiO_2_ flower-like sphere with the petals formed from nanoparticles, mixed with g-C_3_N_4_ sheets structure and nanorod of MWCNT. According to FESEM images of TiO_2_/ SiO_2_/g-C_3_N_4_/MWCNT composite, the distribution of MWCNT in the composite can help to obtain better mechanical strength properties and create strong bonds of MWCNT with other components. Also, the MWCNT acts as a bridge to provide the transport of charge carriers across the pores. The incorporation of MWCNTs leads to a reduction in pore volume, thereby promoting a more compact microstructure and facilitating more efficient charge carrier transport, which collectively enhance the photocatalytic performance^19–21^.

**Fig. 2:**
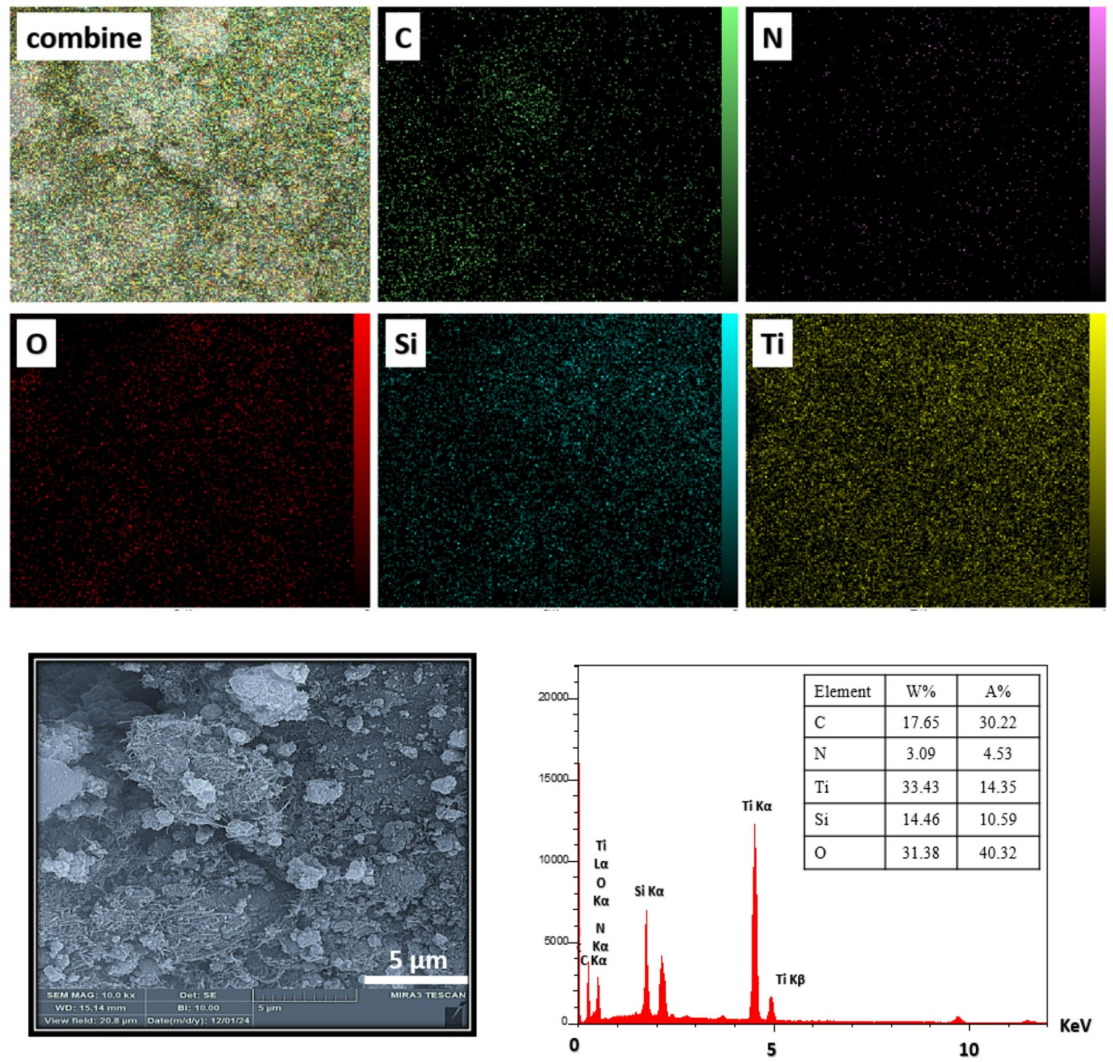
The elemental distribution and EDX spectrum of TiO_2_/ SiO_2_/g-C_3_N_4_/MWCNT composite.

The recognition and spatial distribution of the elements in the TiO_2_/SiO_2_/g-C_3_N_4_/MWCNT composite (containing 11 wt% MWCNT) were analyzed via EDX and elemental mapping, as illustrated in Fig. 2. The EDX spectrum confirmed the presence of carbon (C), nitrogen (N), titanium (Ti), oxygen (O), and silicon (Si) in the composite. Elemental mapping further validated the uniform distribution of C and N, originating from g-C_3_N_4_ and MWCNTs, as well as Ti, Si, and O, which are attributed to the TiO_2_/SiO_2_ hybrid phase. These results confirm the successful integration and homogeneous dispersion of all constituent components within the ternary photocatalyst matrix.

**Fig. 3:**
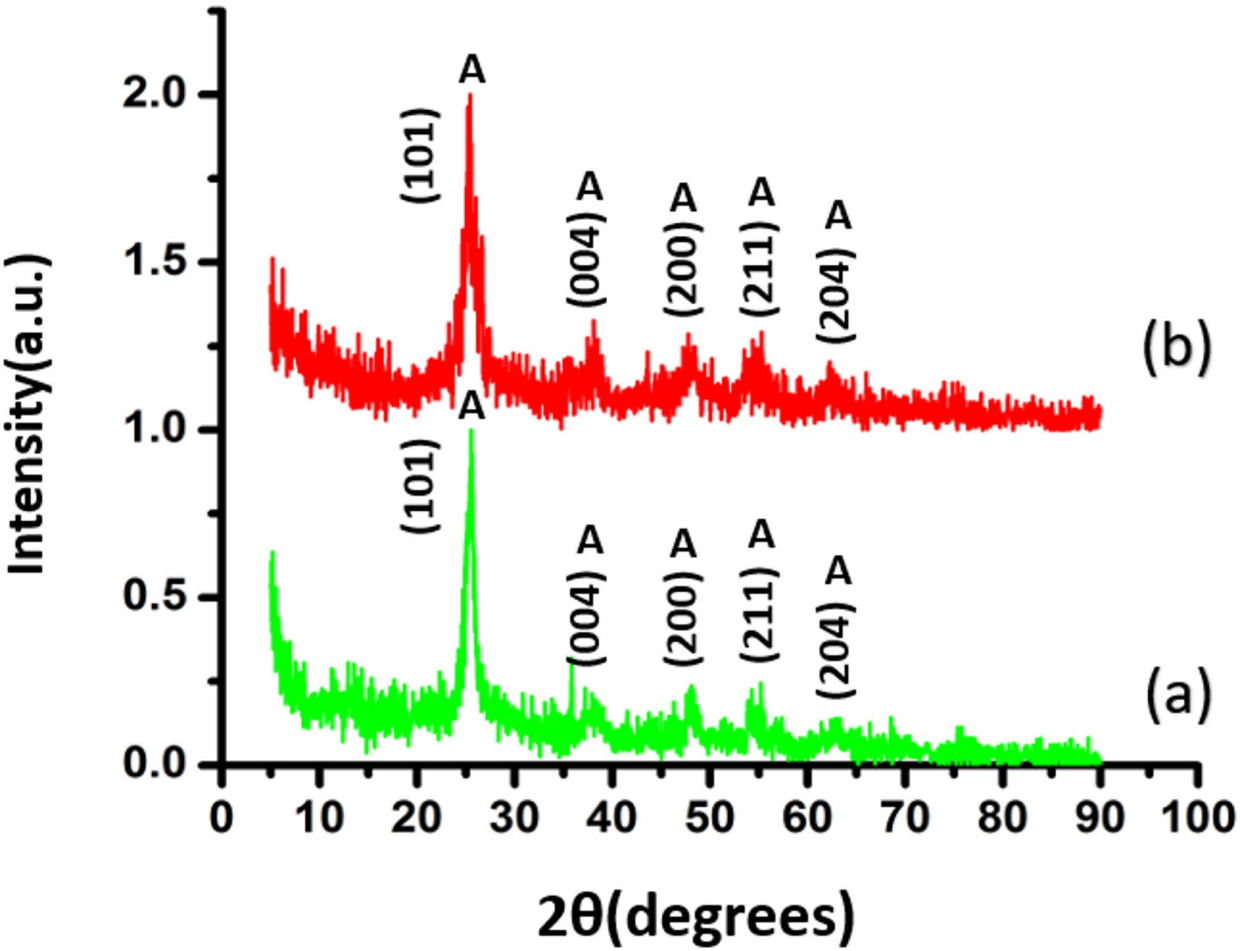
Normalized XRD patterns of (**a**) TiO_2_/ SiO_2_/g-C_3_N_4_ and (**b**) TiO_2_/SiO_2_/g-C_3_N_4_/MWCNT composites.

Figure 3 displays the XRD patterns of TiO_2_/SiO_2_/g-C_3_N_4_ and TiO_2_/SiO_2_/g-C_3_N_4_/MWCNT composites. Both patterns confirm crystallization into the anatase phase, characteristic of the TiO_2_ component, with diffraction peaks aligning well with the standard JCPDS card No. 96-900-9087. As shown in Table 1, the incorporation of MWCNTs has minimal impact on the crystallite size, crystalline structure, and phase composition of the TiO_2_/SiO_2_/g-C_3_N_4_ matrix. Due to the relatively low content of MWCNTs, their characteristic diffraction peaks are not clearly observable in the composite spectrum^12^.

Notably, a slight shift of diffraction peaks toward lower angles was detected, indicating an increase in interplanar spacing and the presence of micro strain within the lattice^19^. The average crystallite sizes, calculated via the Debye–Scherrer equation:

1 $$\:D=\frac{k\lambda\:}{\beta\:\mathrm{cos}\theta\:}$$

where *k* is the shape factor (0.9), *λ* is the X-ray wavelength (1.54060 Å), *β* is the full width at half maximum (FWHM), and *θ* is the Bragg angle. The FWHM of the main peak (101) was 0.3840 for TiO_2_/SiO_2_/g-C_3_N_4_ and 0.3833 for TiO_2_/SiO_2_/g-C_3_N_4_/MWCNT. The crystallite sizes were calculated to be 21.33 nm for the TiO_2_/SiO_2_/g-C_3_N_4_ sample and 21.37 nm for the TiO_2_/SiO_2_/g-C_3_N_4_/MWCNT sample, respectively.

The induced lattice strain (ε) was evaluated using the Stokes–Wilson equation^19^:

2 $$\:\epsilon\:=\frac{\beta\:}{4\mathrm{tan}\theta\:}$$

Upon MWCNT incorporation, the strain increased slightly from 7.28 × 10^−3^ to 7.80 × 10^−3^, suggesting minor lattice distortion induced by carbon nanotube integration^19^.

**Table 1:** The XRD data of TiO_2_/SiO_2_/g-C_3_N_4_ and TiO_2_/SiO_2_/g-C_3_N_4_/MWCNT composites.

Sample	2θ (°)	a = b (Å)	c (Å) d (Å)	Crystallite size (nm)
TiO_2_/SiO_2_ /g-C_3_N_4_	25.51	3.74	9.44 3.48	21.33
TiO_2_/SiO_2_ /g-C_3_N_4_/MWCNT	25.33	3.78	9.44 3.51	21.37

### Chemical structure and bonds of composites

**Fig. 4:**
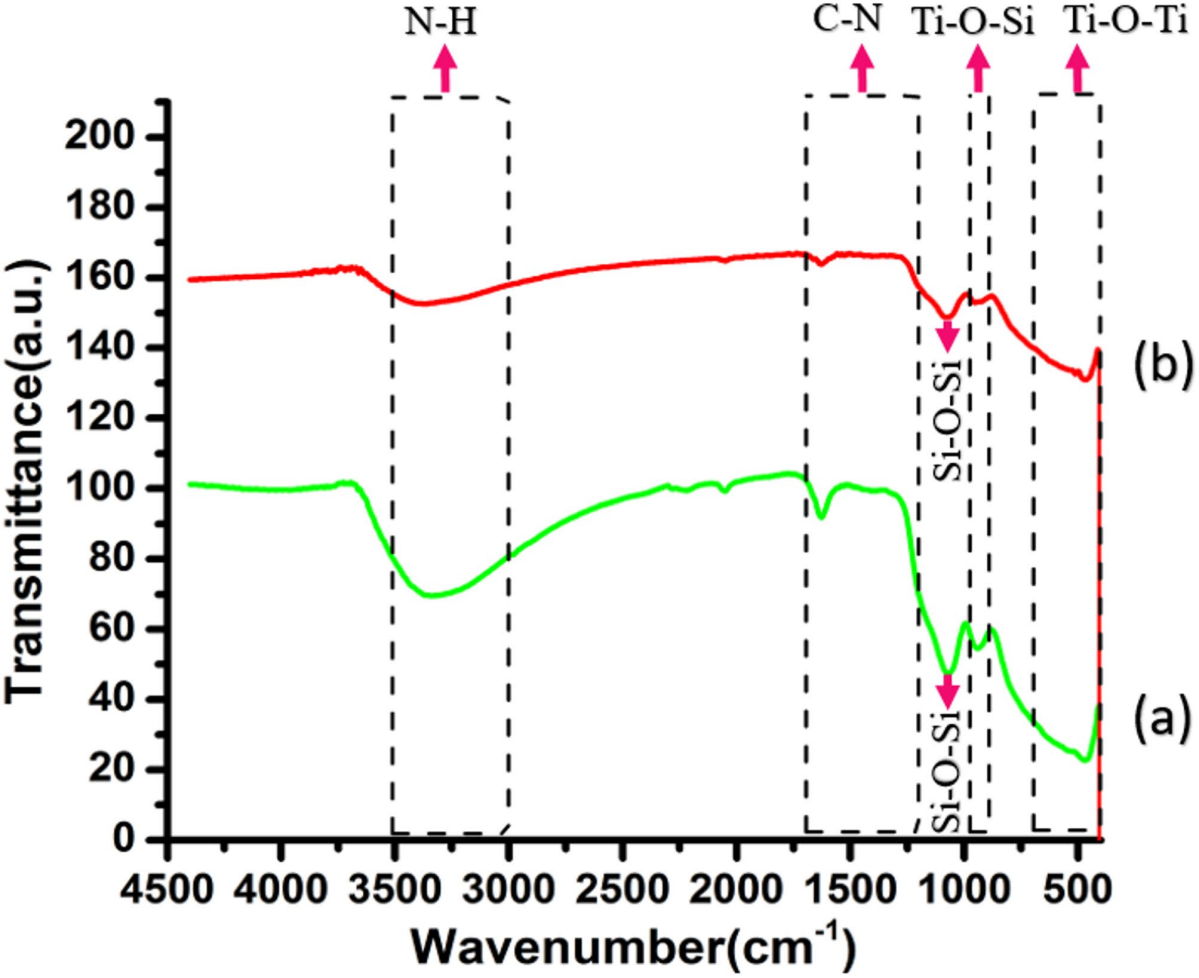
The FTIR spectra of (**a**) TiO_2_/SiO_2_/g-C_3_N_4_ and (**b**) TiO_2_/SiO_2_/g-C_3_N_4_/MWCNT samples.

The chemical structures of TiO_2_/SiO_2_/g-C_3_N_4_ and TiO_2_/SiO_2_/g-C_3_N_4_/MWCNT photocatalysts were investigated using FT-IR spectroscopy, as illustrated in Fig. 4. In the TiO_2_/SiO_2_/g-C_3_N_4_ composite, characteristic vibrations in the range of 400–700 cm^−1^ were attributed to Ti–O–Ti stretching modes^22^. A broad absorption band between 3000 and 3500 cm^−1^ corresponds to N–H stretching vibrations^23^. The presence of g-C_3_N_4_ was confirmed by distinct peaks near 1200 cm^−1^ and 1650 cm^−1^, which are attributed to the stretching vibrations of C–N heterocyclic structures^24^. A sharp peak in the range of 920–965 cm^−1^ is indicative of Ti–O–Si stretching modes, confirming the successful incorporation of SiO_2_ into the TiO_2_ matrix^25^. Additionally, a band around 1100 cm^−1^ was associated with asymmetric Si–O–Si stretching vibrations^14^. In the FT-IR spectrum of the TiO_2_/SiO_2_/g-C_3_N_4_/MWCNT composite, all prominent bands of the TiO_2_/SiO_2_/g-C_3_N_4_ structure remained intact, indicating stable chemical bonding. The absorption features associated with MWCNTs overlapped with the C–N heterocycle region, suggesting successful integration of MWCNTs into the nanocomposite framework^12^. These results collectively confirm the formation of a well-structured, chemically bonded ternary composite.

The MWCNTs in the TiO_2_/SiO_2_/g-C_3_N_4_/MWCNT composite can physically and electronically interact with the other components by occupying pores and interstitial voids, as observed in FESEM images. This integration not only enhances interfacial contact but also facilitates efficient charge transfer among the composite constituents. As a result of these interactions, the electronic environments of various functional groups are altered, potentially leading to shifts in vibrational frequencies and suppression of peak intensities in the FT-IR spectra. Such changes suggest strong bonding or hybridization effects between MWCNTs and the surrounding matrix, further confirming the formation of an intimately connected nanocomposite structure^26^.

### Optical properties

**Fig. 5:**
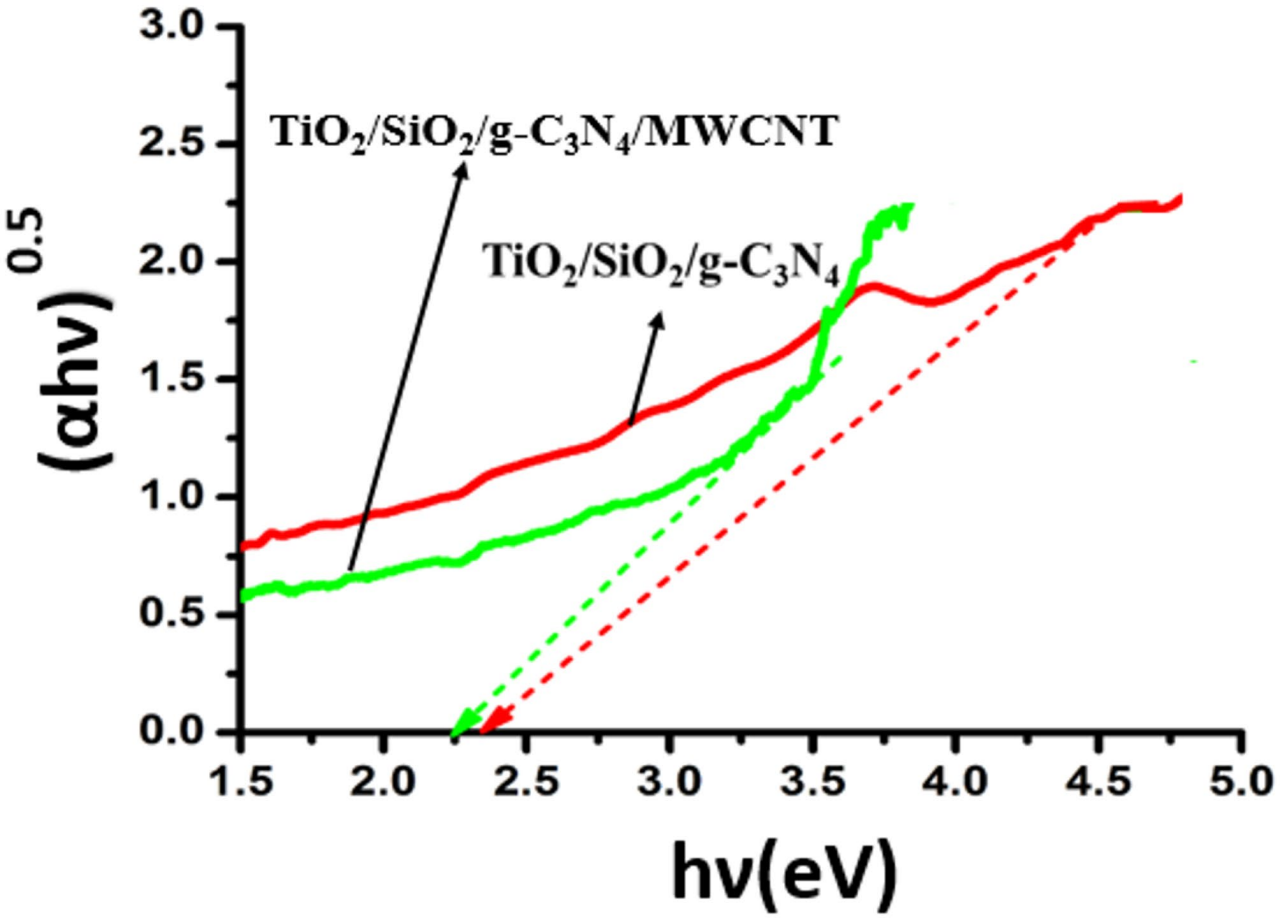
Plot of (αhν)^0.5^ versus photon energy for TiO_2_/ SiO_2_/g-C_3_N_4,_ and TiO_2_/ SiO_2_/g-C_3_N_4_/MWCNT samples.

The optical properties and band gap energy of TiO_2_/ SiO_2_/g-C_3_N_4_ and TiO_2_/SiO_2_/g-C_3_N_4_/MWCNT were studied in Fig. 5. The band gap values of TiO_2_/SiO_2_/g-C_3_N_4_ and TiO_2_/SiO_2_/g-C_3_N_4_/MWCNT were calculated about 2.37 and 2.25 eV, respectively by using Eq. (3) and plot of (αhν)^1/2^ versus hν. As shown in Fig. 5, MWCNT reduces the optical band gap and improves the adsorption of visible light. Reduction of band gap means generation of charge carriers by low energy excitation. Also, reduction in band gap of TiO_2_/SiO_2_/g-C_3_N_4_/MWCNT sample compared to TiO_2_/SiO_2_/g-C_3_N_4_ can show the role of MWCNT as a visible light sensitizer^12^. The narrowing of the band gap energy of TiO_2_/SiO_2_/g-C_3_N_4_/MWCNT composite and adsorption of light in longer wavelength by this composite is related to the interaction between MWCNT with other components (TiO_2_/SiO_2_ and g-C_3_N_4_) and bonding among them for the formation of defect-induced energy states within the band gap^12,19,20^.

3 $${(\alpha {\rm{h}}\nu {\rm{)}}^{{\rm{1/2}}}}{\rm{ = C}}\left( {{\rm{h}}\nu - {{\rm{E}}_{\rm{g}}}} \right)$$

where h is Planck’s constant and ν is the frequency of light. α and E_g_ are the absorption coefficient and the band gap energy, respectively^27^.

The band structure diagram for TiO_2_/SiO_2_/g-C_3_N_4_/MWCNT composite can be investigated by VB XPS spectrum. We estimated the valence band (VB) position of TiO_2_/SiO_2_ sample as ~ 2.90 eV using VB XPS analysis in our previous work^15^. Using the value of 2.87 eV for the band gap energy, the conduction band (CB) position was calculated about 0.03 eV^15,28^. Coupling of TiO_2_/SiO_2_ hybrid, g-C_3_N_4_ and MWCNT results in forming heterojunctions which have important roles in separation of charge carriers and their transport. Therefore, better photocatalytic activity of TiO_2_/SiO_2_/g-C_3_N_4_/MWCNT composite can be expected^29,30^.

**Fig. 6:**
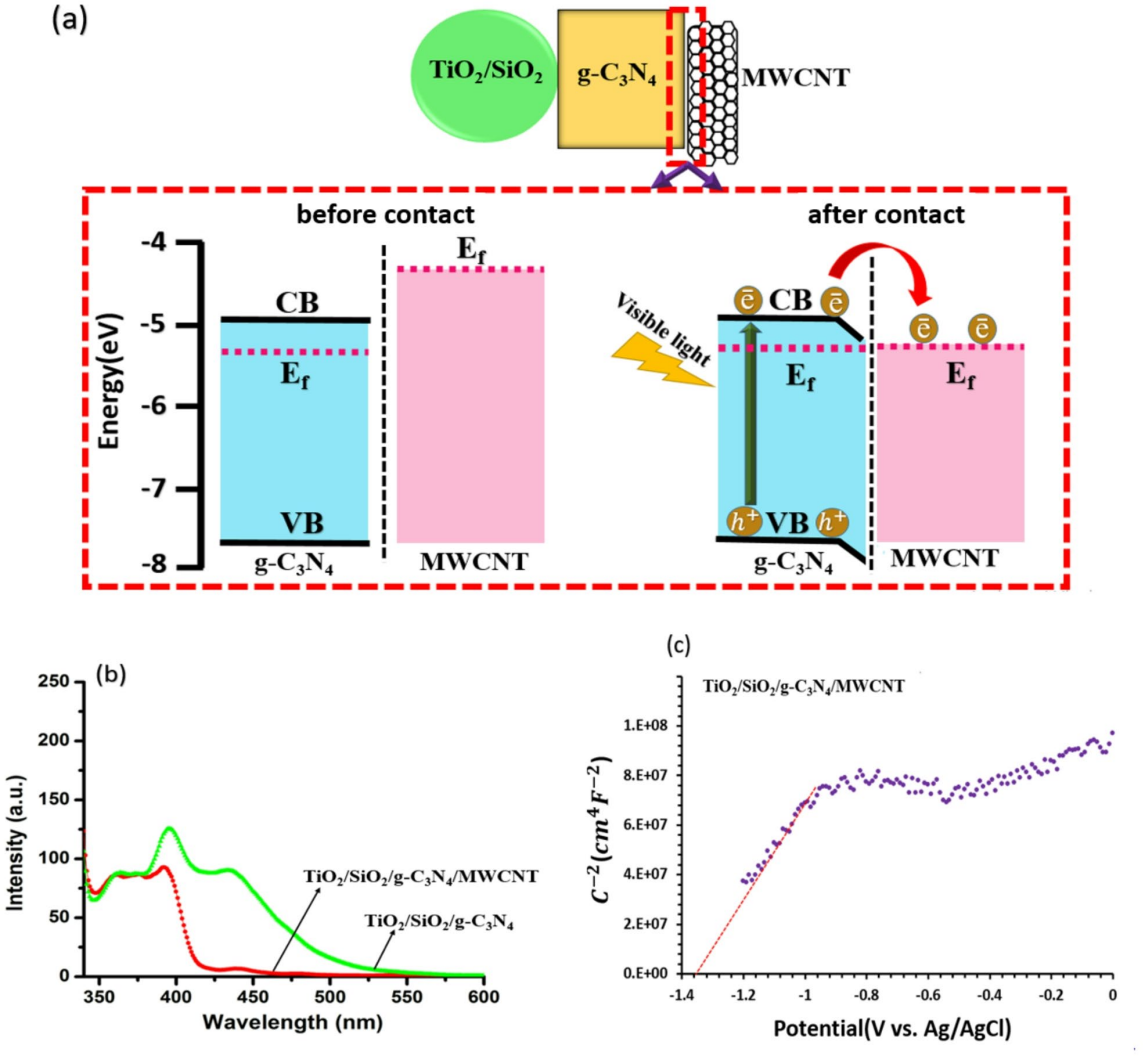

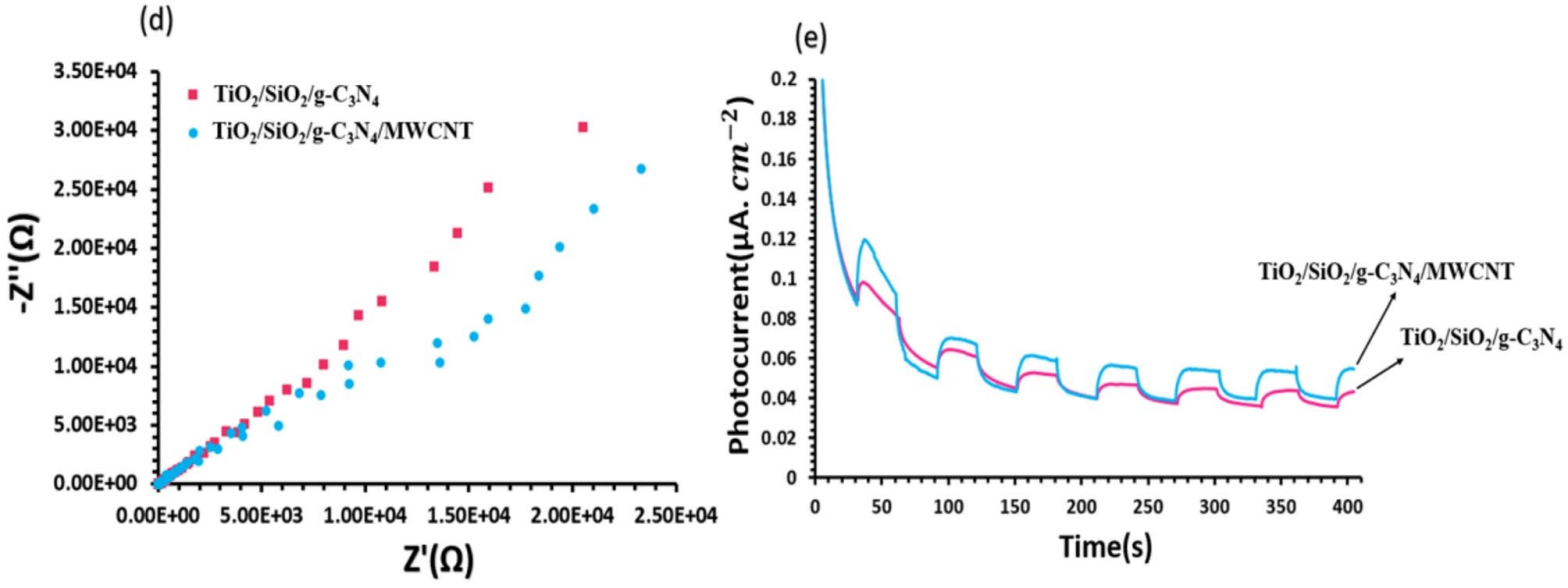
(**a**) The band structure diagram and (**b**) PL spectra of TiO_2_/SiO_2_/g-C_3_N_4,_ and TiO_2_/SiO_2_/g-C_3_N_4_/MWCNT samples (**c**) Mott–Schottky curve of TiO_2_/SiO_2_/g-C_3_N_4_/MWCNT sample (**d**) electrochemical impedance spectroscopy and (**e**) transient photocurrent plots of TiO_2_/ SiO_2_/g-C_3_N_4,_ and TiO_2_/SiO_2_/g-C_3_N_4_/MWCNT samples.

Considering the obtained results and literature^31^, a band structure diagram for better comprehension of the photocatalyst performance was proposed as shown in Fig. 6a. Under visible light radiation, the photogenerated electrons in the CB of TiO_2_/SiO_2_ recombine with holes in the VB of g-C_3_N_4_. Therefore, there are electrons in the CB of g-C_3_N_4_ and holes in the VB of TiO_2_/SiO_2_ which have potential to produce active radicals for degradation of pollutants^15^. with addition of MWCNTs, mixed dimensional van der Waals heterojunctions form between g-C_3_N_4_ sheets and MWCNT nanotubes^31^. When g-C_3_N_4_ and MWCNTs are in contact, their fermi levels (E_f_) tend to equilibrate due to interfacial charge redistribution. The work function of MWCNTs has been reported to lie in the range of − 4.33 to − 4.95 eV^12,19,20,31^. After contact band bending occurs toward the MWCNT. The MWCNTs serve as electron reservoirs, suppressing recombination and thereby enhancing the separation efficiency of photogenerated electrons and holes upon light exposure^12,31^.

For revealing the separation efficiency and migration of photogenerated carriers, PL spectroscopy as a highly dependent technique on recombination of free carriers was performed, as shown Fig. 6b. Two peaks are observed; the first peak in the UV region located at ~ 390 nm is related to the recombination of excitons near the band-edge and the second wide peak in the visible region at approximately 440 nm is attributed to the presence of surface trap states^12,31^. These peaks originate from TiO_2_ and g-C_3_N_4_ components. SiO_2_ and MWCNT do not contribute to PL directly but influence the emission intensities by modifying surface states and facilitating charge transfer. The PL spectra of both samples exhibit nearly identical peak positions; however, a pronounced decrease in emission intensity is observed upon the incorporation of MWCNTs. With the addition of MWCNTs the ratio of visible to UV emission intensity (I_Vis_/I_UV_) decreases significantly from 0.71 to 0.07, indicating an enhancement in the separation efficiency of photoinduced charge carriors. This suppression of charge carrier recombination is primarily due to the formation of local heterojunctions among MWCNTs, TiO_2_/SiO_2_ hybrids, and g-C_3_N_4_ sheets. As a result of the improved charge separation, the carriers exhibit prolonged lifetimes and are effectively transferred to active sites on the photocatalyst surface, thereby can enhance the overall photocatalytic activity^12,15^.

Mott–Schottky (M–S) analysis provides useful information about flat-band potential (E_FB_) of a photocatalyst, carrier density, and band-edge positions, parameters that are fundamental for evaluating charge separation, photocatalytic performance, and redox feasibility. The M-S curve was indicated in Fig. 6c. Considering the M-S curve of the TiO_2_/SiO_2_/g-C_3_N_4_/MWCNT photocatalyst, the positive linear slope (1.68 × 10^8^ F^− 2^ cm^4^ V^− 1^) confirms n-type behavior and yields E_FB_ = − 1.36 V (vs. Ag/AgCl). Converting to standard scales gives E_FB_ = − 1.16 V (vs. NHE). The position of the conduction band of n-type semiconductor is considered to be close to E_FB_, indicating a highly reducing conduction band suitable for generating .O_2_^**−**^ and driving pollutant degradation. According to the obtained results, the position of the conduction band is governed by g-C_3_N_4_ under the Z-scheme charge transfer mechanism^32,33^.

To provide mechanistic data of the TC photodegradation process by the synthesized photocatalysts, electrochemical impedance spectroscopy (EIS) and transient photocurrent measurments for the TiO_2_/SiO_2_/g-C_3_N_4_ samples with and without MWCNTs were performed. Figure 6d shows electrochemical impedance spectrograms of the samples. Since arc radius corresponds to charge transfer layer resistance^34^, smaller arc radius for the TiO_2_/SiO_2_/g-C_3_N_4_/MWCNT composite indicates lower layer resistance against charge transfer. Then, higher efficiency for the photoinduced electron–hole separation and transfer, which enhance the photocatalytic activity, is deduced with the presence of MWCNTs in the TiO_2_/SiO_2_/g-C_3_N_4_ ternary composite. These results are in agreement with the results derived from PL spectra (Fig. 6b).

The transient photocurrent response is used for a qualitative evaluation of photo-induced charge separation and recombination in the photocatalytic process. Figure 6e represents the transient variation in current of the samples by turning on and off the illumination. The stronger photocurrent of the TiO_2_/SiO_2_/g-C_3_N_4_/MWCNT composite shows that photoexcited charge carriers are effictively separated and participate in the reaction. The results suggest that the introduction of MWCNTs into the composite improves the separation efficiency and transfer of photogenerated electrons and holes through formation of so many local heterojunctions^33^.

### The BET surface area measurement of composites

Since surface area plays important role in the photocatalytic process, BET analysis was performed. Table 2 presents the data obtained from BET analysis. It is observed that the surface area, total volume and mean diameter of the pores in the TiO_2_/SiO_2_/g-C_3_N_4_ composite reduce with addition of MWCNT^19,20^. However, this reduction is not considerable to affect the photocatalytic activity severely. Although the BET surface area decreases slightly with MWCNT incorporation, the nanotubes enhance adsorption efficiency by introducing functional groups and π–π interactions while simultaneously preventing particle agglomeration and optimizing pore accessibility^35^.

**Table 2:** The textural parameters of the TiO_2_/SiO_2_/g-C_3_N_4_ and TiO_2_/SiO_2_/g-C_3_N_4_/MWCNT composites.

Sample	BET surface area (m^2^/g)	Total pore volume (cm^3^/g)	Mean pore diameter (nm)
TiO_2_/SiO_2_ /g-C_3_N_4_	224.83	0.33	5.89
TiO_2_/SiO_2_ /g-C_3_N_4_/MWCNT	215.85	0.31	5.75

### Photodegradation of MB

**Fig. 7:**
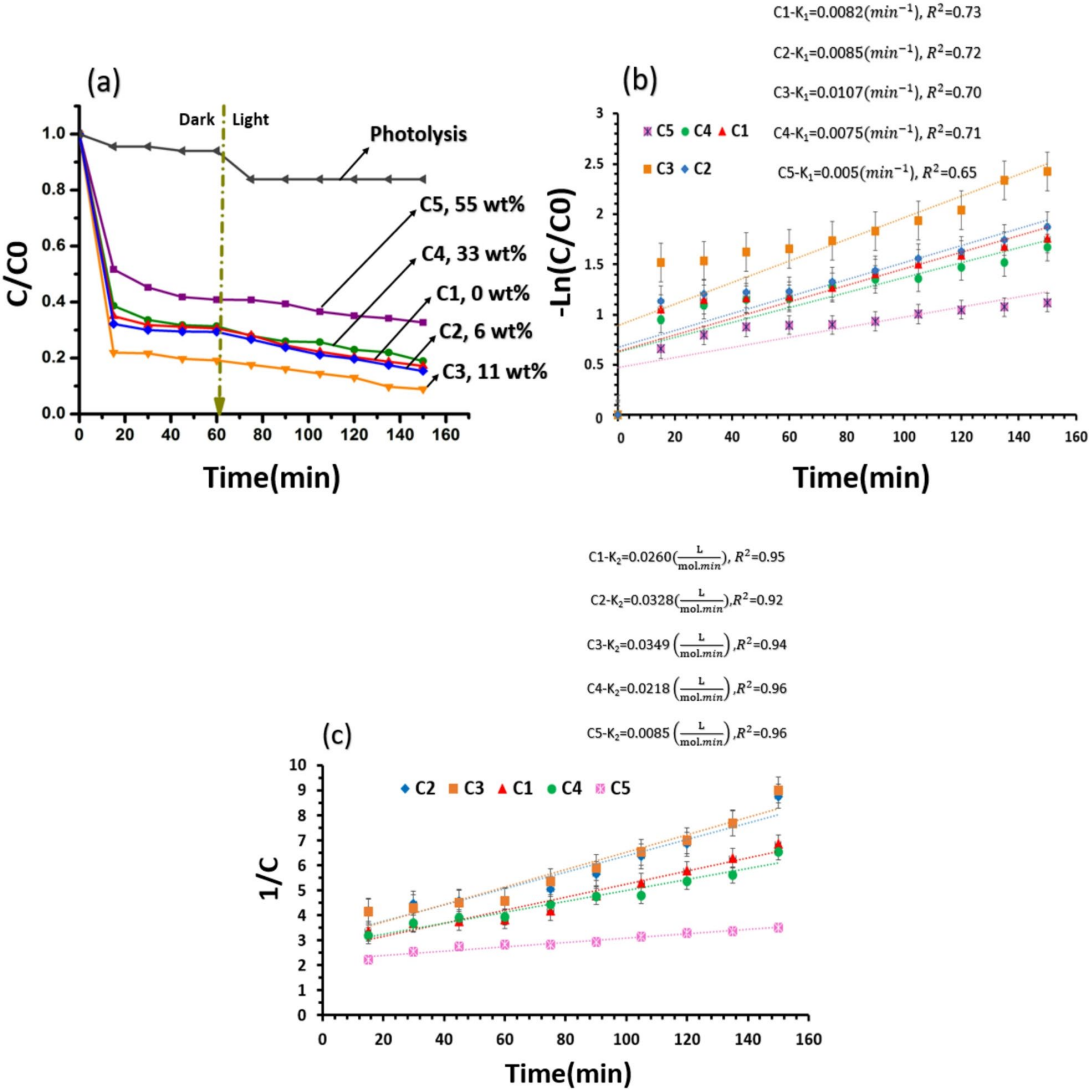
(**a**) The photocatalytic activity and photocatalytic degradation rate by (**b**) the first order kinetic model and (**c**) the second order kinetic model using TiO_2_/SiO_2_/g-C_3_N_4_ as C1, and TiO_2_/SiO_2_/g-C_3_N_4_/MWCNT with different concentrations of MWCNTs denoted as C_2_-C_5_.

The Photocatalytic capability of TiO_2_/SiO_2_/g-C_3_N_4,_ and TiO_2_/SiO_2_/g-C_3_N_4_/MWCNT with different concentrations of MWCNT was determined by a standard dye of MB with concentration of 20 mg/l under visible light irradiation. The effect of the MWCNT amount in the TiO_2_/SiO_2_/g-C_3_N_4_ composite on the photocatalytic removal of MB has been depicted Fig. 7. As the concentration of MWCNT increased from 0 to 11 wt%, the photodegradation efficiency of MB (i.e., [(C0-C)/C0]×100, where C0 and C are the initial and final concentrations^36^ rose from 83% to 92% in 150 min. Figure 7a shows that TiO_2_/SiO_2_/g-C_3_N_4_ composite without and with 11 wt% MWCNT decolorize 83% and 92% dye in 150 min, respectively. This enhancement in the photocatalytic reaction with proper concentration of MWCNT was attributed to charge separation efficiency through the formation of heterojunction at the interface and enhancement of the visible light absorption due to presence of MWCNTs^12^. Actually, MWCNTs act as electron reservoirs to reduce the recombination rate of the photogenerated electron-hole pairs resulting in increase of photocatalytic activity^12^. Notable, dye decoloration decreases with enhancing the MWCNT amount. This result indicates that an excess amount of MWCNT can prohibit the active sites on the composite surface from absorption of visible light and adsorption of pollutants^12,19^. It seems that the photocatalytic activity increases with the addition of MWCNT and decreases with further increase in MWCNT, which could be due to the reduction of the effective surface area with the presence of MWCNT. After 30%, the reduction in the specific surface area is greater and the contribution of the composite to the photocatalytic activity decreases due to the covering of the surface-active sites with MWCNT. To determine the dominant kinetic model of the degradation process, -Ln(C/C_0_) vs. time for the pseudo-first order kinetic model (− Ln(C/C_0_) = k_1_t) and 1/C vs. time for the second order kinetic model (i.e., (1/C)=(1/C_0_)+k_2_t) were plotted as indicated in Fig. 7b,c. The linear plot with a good correlation coefficient (R^2^ close to 1) in Fig. 7c shows the degradation follows second order kinetic model^20^. The excellent photocatalytic activity is observed for TiO_2_/SiO_2_/g-C_3_N_4_/MWCNT-11wt% sample.

### Photodegradation of TC in two water systems

In order to investigate the influence of temporary water hardness on photocatalytic degradation efficiency, comparative experiments were conducted using TiO_2_/SiO_2_/g-C_3_N_4_ and TiO_2_/SiO_2_/g-C_3_N_4_/MWCNT photocatalysts for the degradation of TC. The photocatalytic tests were performed in both deionized (pure) water and water containing CaCO_3_. The presence of CaCO_3_ simulates temporary hardness by introducing carbonate species into the aqueous system. The ionic speciation is highly pH-dependent: at neutral pH (∼7), bicarbonate (HCO_3_^−^) dominates, while at alkaline pH (> 10), carbonate (CO_3_^2−^) becomes the predominant species^17^.

**Fig. 8:**
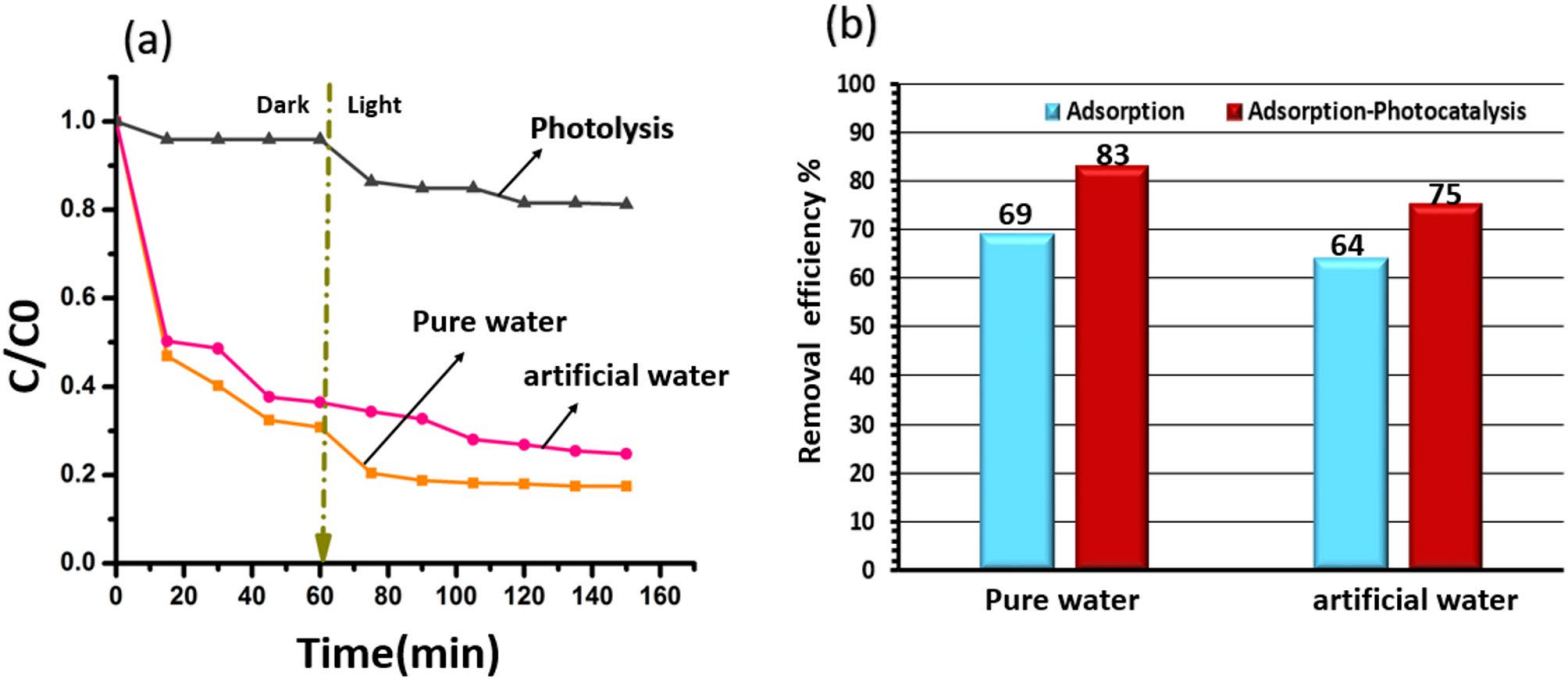
(**a**) The adsorption in the dark and photocatalysis under visible light for the removal of TC with concentration of 20 mg/l and (**b**) the adsorption efficiency (%) in the dark and adsorption-photocatalysis efficiency (%) for the removal of TC with concentration of 20 mg/l using TiO_2_/SiO_2_/g-C_3_N_4_ catalyst in pure water and water containing CaCO_3_.

The photocatalytic degradation of TC by TiO_2_/SiO_2_/g-C_3_N_4_ was evaluated in both deionized water and bicarbonate-rich water (pH ≈ 7, containing HCO_3_^−^ ions), as depicted in Fig. 8. The results show that the TiO_2_/SiO_2_/g-C_3_N_4_ composite achieved removal efficiencies of 83% in pure water and 75% in CaCO_3_-containing water after 150 min. The reduced performance in hard water is attributed to the interference of bicarbonate ions, which inhibit pollutant adsorption and limit interaction with the photocatalyst surface^13,18^.

**Fig. 9:**
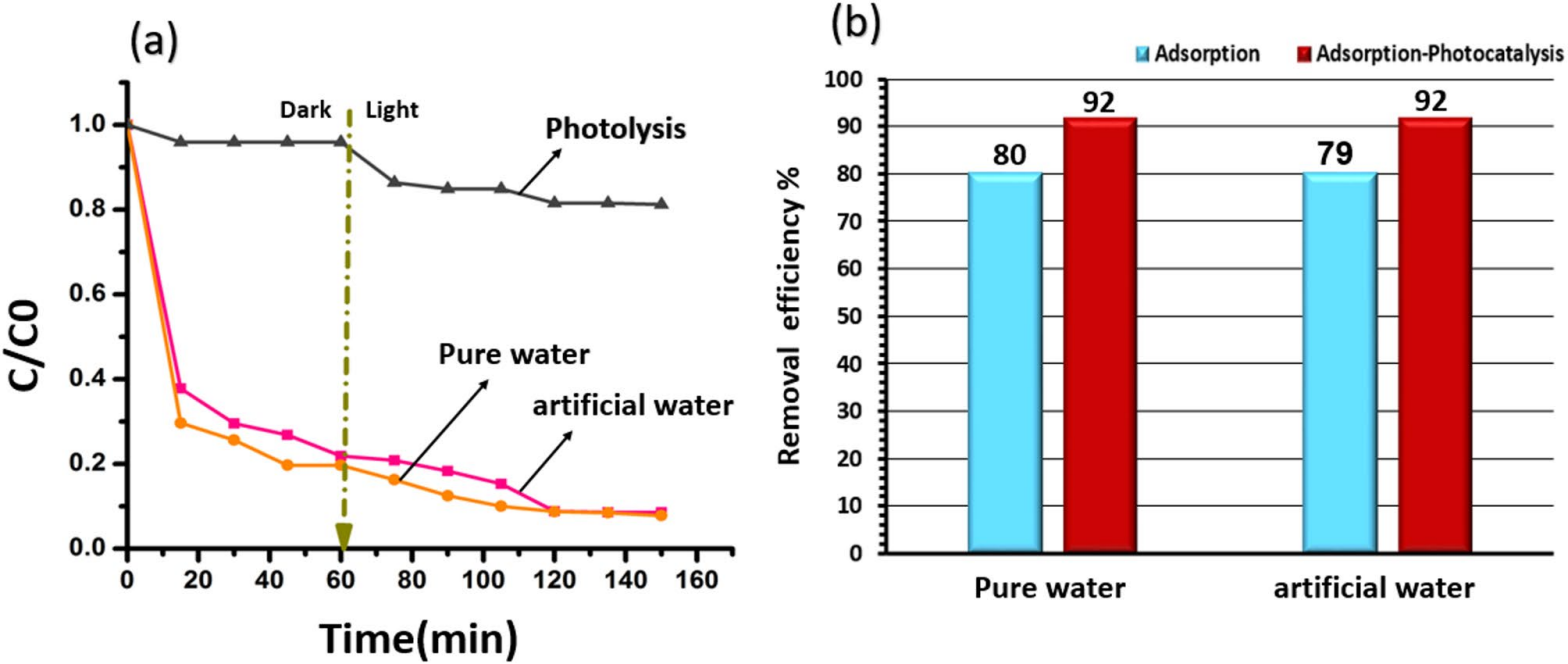
(**a**) The adsorption in the dark and photocatalysis under visible light for the removal of TC with concentration of 20 mg/l and (**b**) the adsorption efficiency (%) in the dark and adsorption-photocatalysis efficiency (%) for the removal of TC with concentration of 20 mg/l using TiO_2_/SiO_2_/g-C_3_N_4_/MWCNT catalyst in pure water and water containing CaCO_3_.

In contrast, Fig. 9 demonstrates that the TiO_2_/SiO_2_/g-C_3_N_4_/MWCNT composite maintained high photocatalytic activity under the same conditions, achieving 92% degradation efficiency in both pure and bicarbonate-containing water after 150 min. This stability is attributed to the exceptional charge transfer and separation capabilities of MWCNTs, which minimize electron–hole recombination and enhance the formation of ^·^OH radicals. These radicals can oxidize HCO_3_⁻ to generate carbonate radicals (CO_3_^·−^), as described in Eq. (4), which contribute to the degradation of organic pollutants^17^.

4 $$\:{\mathrm{H}\mathrm{C}\mathrm{O}}_{3}^{-}+{\mathrm{O}\mathrm{H}}^{\bullet\:}\to\:{\mathrm{C}\mathrm{O}}_{3}^{\bullet\:-}+{\mathrm{H}}_{2}\mathrm{O}$$

In summary, in CaCO_3_-rich water, both Ca^2+^ and HCO_3_^−^ ions adversely influence the photocatalytic performance of the TiO_2_/SiO_2_/g-C_3_N_4_ composite. Adsorption of Ca^2+^ ions onto the photocatalyst surface blocks active sites and thereby diminishes the adsorption of TC molecules. Meanwhile, HCO_3_^−^ acts as a scavenger of ^·^OH radicals and competes with TC for reactive species, leading to lower degradation efficiency in hard water. In the TiO_2_/SiO_2_/g-C_3_N_4_/MWCNT composite, the presence of MWCNT suppressing electron-hole recombination. The efficient charge separation facilitates the generation of ^·^OH radicals, which subsequently react with HCO_3_^−^ ions to yield CO_3_^·−^ radicals. These reactive species contribute to the degradation of TC, enabling the TiO_2_/SiO_2_/g-C_3_N_4_/MWCNT composite to sustain high degradation efficiency under both conditions^17,18,37,38^.

**Fig. 10:**
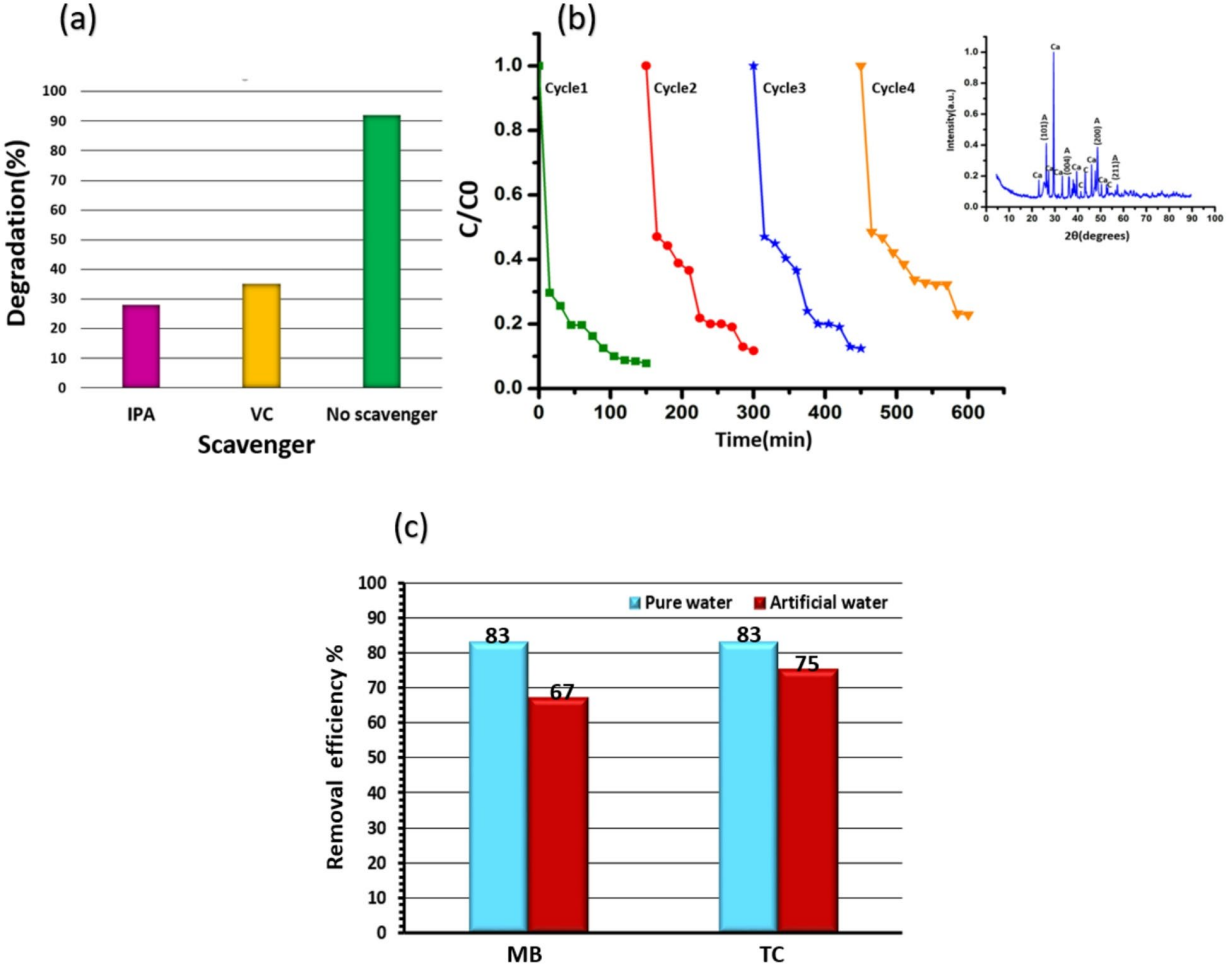
(**a**) Photodegradation of TC with concentration of 20 mg/l under visible light with radical scavengers (**b**) cycle runs of TC degradation with concentration of 20 mg/l in water containing CaCO_3_ under visible light. Inset shows XRD analysis of composite after four cycle runs of TC degradation (**c**) the adsorption-photocatalysis efficiency (%) for the removal of MB and TC with concentration of 20 mg/l using TiO_2_/SiO_2_/g-C_3_N_4_ catalyst in pure water and water containing CaCO_3_.

To identify the main surface species involved in the photocatalytic degradation of TC over the TiO_2_/SiO_2_/g-C_3_N_4_/MWCNT sample under visible light irradiation, two scavengers, isopropanol (IPA) and ascorbic acid (Vitamin C), were employed. The roles of ^·^OH radicals and ^·^O_2_^**−**^ radicals in the degradation process were determined using IPA and VC, respectively^39,40^, as shown in Fig. 10a. In the presence of the scavengers (1 mM), the photodegradation of TC (20 mg/L) under visible light was evaluated. Upon the addition of IPA and VC, the degradation efficiency decreased from 92% to approximately 28% and 35%, respectively, confirming that ^·^OH radicals and ^.^O_2_^**−**^radicals are the dominant reactive species in the photocatalytic degradation of TC by the TiO_2_/SiO_2_/g-C_3_N_4_/MWCNT nanostructures.

For confirming the practical relevance of the synthesized photocatalyst, photostability and reusability of TiO_2_/SiO_2_/g-C_3_N_4_/MWCNT sample were studied during four 150-min photocatalytic cycling tests in Fig. 10b. The photodegradation efficiency of TC exhibits a gradual decline, primarily attributed to the loss of photocatalyst during the reaction, cleaning, and drying processes. Nevertheless, after four consecutive cycles, TC degradation still reaches 77% within 150 min. Comparison of the XRD pattern in the inset of Fig. 10b with that in Fig. 3b indicates that, although peaks associated with CaCO_3_, a compound typically found in hard water, are present, the structure and anatase phase of TiO_2_ remain intact.

These findings highlight the enhanced applicability of the MWCNT-modified composite in real water systems with temporary hardness. Figure 10c presents a comparative evaluation of TC and MB degradation by the TiO_2_/SiO_2_/g-C_3_N_4_ composite in deionized water and water containing CaCO_3_. The reduction in photocatalytic efficiency observed in hard water is more pronounced for MB than for TC. This difference may be attributed to the relative molecular sizes of the pollutants. TC, possessing a larger molecular diameter than Ca^2+^ and HCO_3_^−^ ions, experiences less steric hindrance during adsorption onto the catalyst surface. Consequently, the presence of ions has a minimal effect on its photocatalytic degradation. In contrast, the molecular size of MB is comparable to that of Ca^2+^ and HCO_3_^−^, allowing these ions to compete more effectively for active sites and thereby hinder interactions between MB molecules and the catalyst surface. As a result, the presence of ionic species significantly suppress the efficiency of MB removal^18^. In brief, larger molecular framework and multifunctional nature of TC make it less susceptible to site competition from Ca^2+^ and HCO_3_^−^, whereas MB, being smaller and cationic, is more strongly influenced by ionic competition^41–43^.

**Fig. 11:**
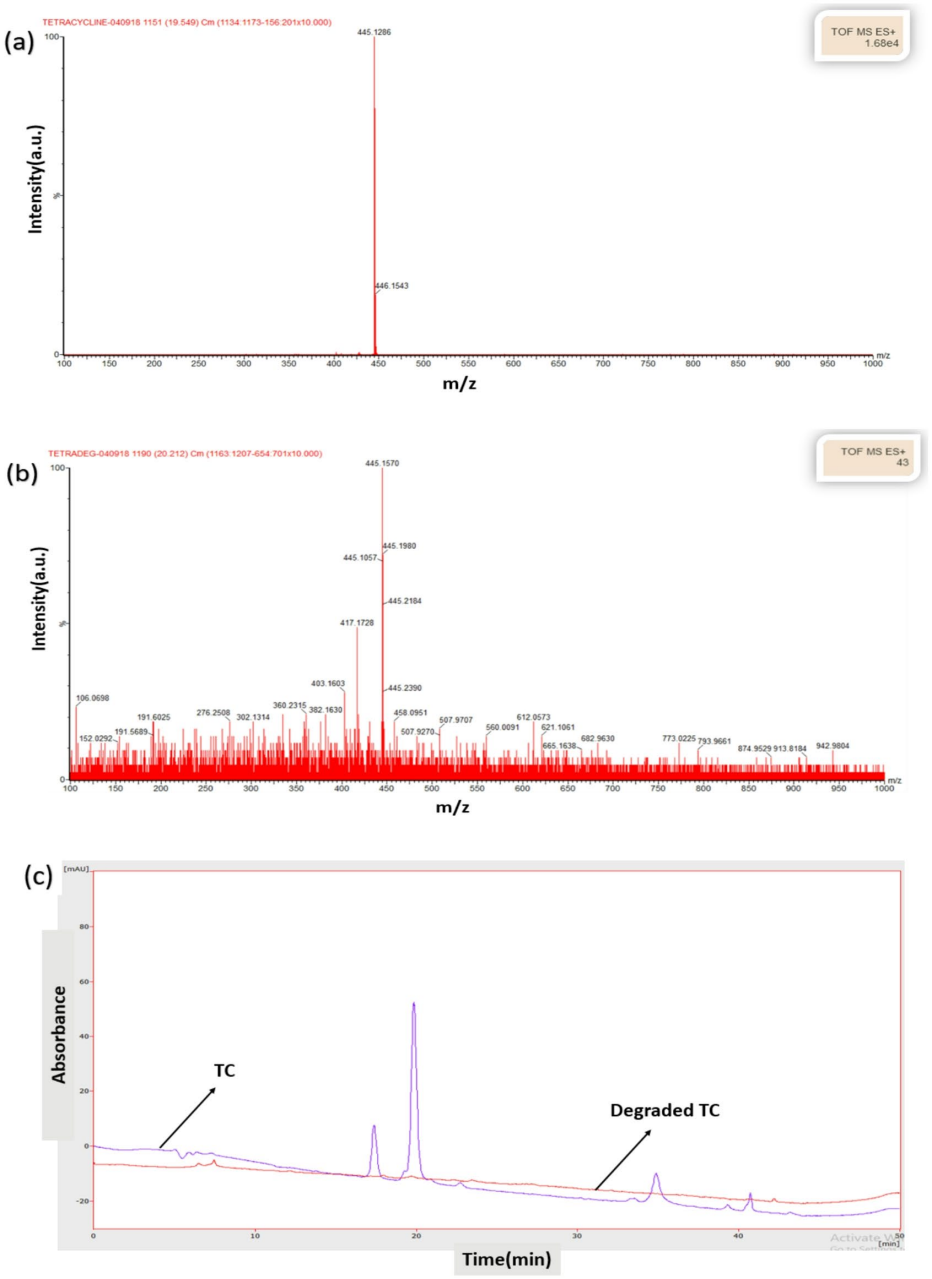
LC-MS chromatograms for (**a**) TC and (**b**) degraded TC samples (**c**) transient curve of the samples.

Photocatalytic degradation products of TC were analyzed by liquid chromatography–mass spectrometry (LC-MS), as shown in Fig. 11. In the initial TC solution (20 mg/L), a prominent peak at m/z = 445 was observed in Fig. 11a. After 150 min of photocatalytic treatment, the intensity of this peak significantly decreased, indicating extensive degradation of TC (Fig. 11b). Moreover, several minor peaks appeared in the m/z < 425 range, corresponding to smaller and simpler degradation products. These findings confirm complete mineralization and demonstrate the absence of toxic intermediates. Based on Fig. 11c, TC peaks appeared at retention times of approximately 17 and 19 min, whereas no corresponding peaks were detected for the degraded sample^44^.

### Discussion and suggested photocatalytic mechanism

**Fig. 12:**
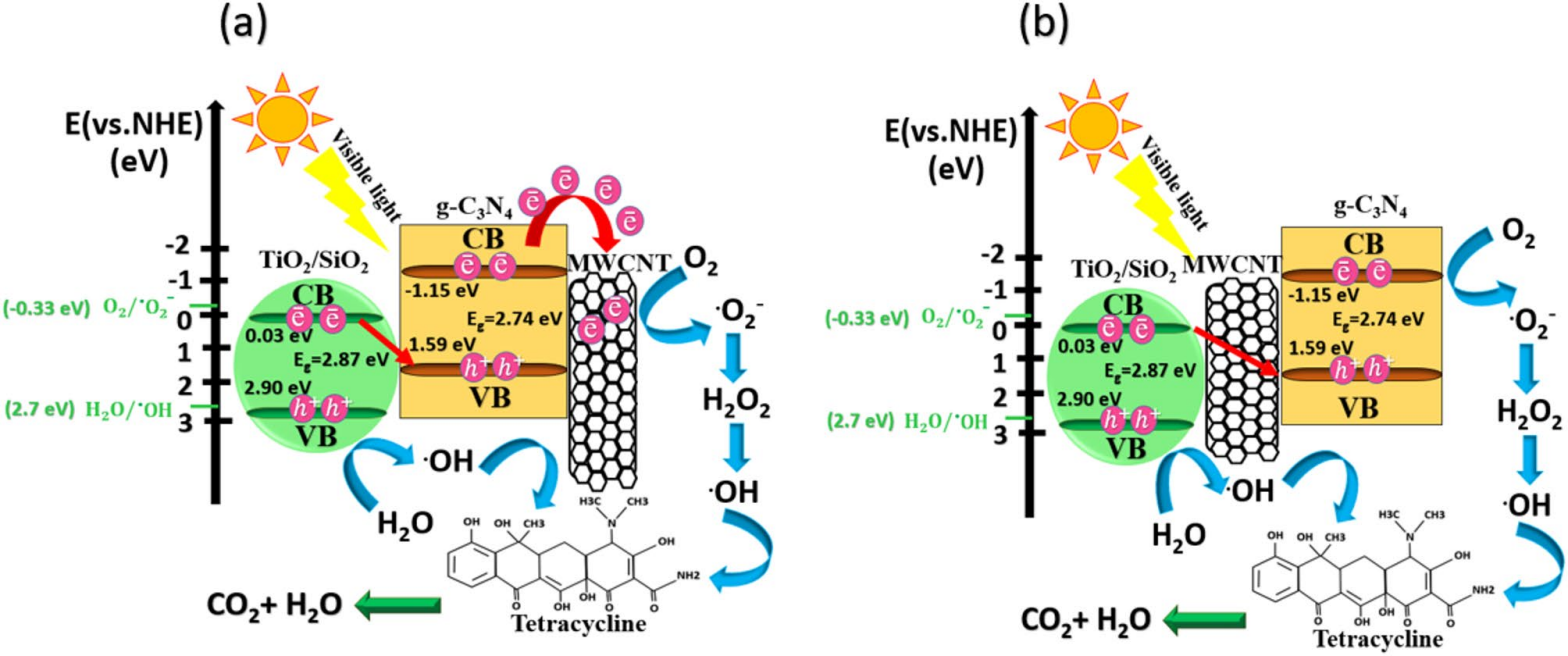
Two photocatalytic mechanisms for charge carrier separation at interfaces under visible light (**a**) MWCNT as a photosensitizer (**b**) MWCNT as a mediator.

The photocatalytic mechanism of TiO_2_/SiO_2_/g-C_3_N_4_/MWCNT composite in Fig. 12 to understand charge carrier separation at interfaces and improvement of photocatalytic efficiency under visible light is suggested. The conduction band minimum (E_CB_) and valence band maximum (E_VB_) of g-C_3_N_4_ were calculated using Eqs. (5) and (6)^45–47^:

5 $${{\rm{E}}_{{\rm{VB}}}} = \chi - {{\rm{E}}_{\rm{e}}} + {\rm{ }}0.{\rm{5}}{{\rm{E}}_{\rm{g}}}$$

6 $${{\rm{E}}_{{\rm{CB}}}} = {{\rm{E}}_{{\rm{VB}}}} - {{\rm{E}}_{\rm{g}}}$$

where $\:{\mathrm{E}}_{\mathrm{g}}$and χ represent the band gap and absolute electronegativity of the semiconductor, respectively, with χ valued at 4.72 eV for g-C_3_N_4_. Additionally, Ee denotes the energy of free electrons on the hydrogen scale, typically taken as 4.5 eV^45–47^. Based on X-ray photoelectron spectroscopy (XPS) data from our previously published work^28^, the VB and CB edge potentials of TiO_2_/SiO_2_ were estimated using Eq. (6) to be 2.90 eV and 0.03 eV, respectively. Similarly, the VB and CB edge potentials for g-C_3_N_4_, calculated using the same approach and input parameters, were determined to be 1.59 eV and − 1.15 eV, respectively^45–47^. Upon visible light irradiation, g-C_3_N_4_ becomes photoactivated, generating electron–hole pairs as electrons are excited from the VB to the CB, leaving behind positively charged holes in the VB. However, these photogenerated holes exhibit limited oxidative ability toward water molecules (H_2_O) and hydroxide ions (OH^−^). This is due to the VB edge potential of g-C_3_N_4_ being only 1.59 eV versus the normal hydrogen electrode (NHE), which is less positive than the redox potentials required for ^·^OH radical formation—specifically, E⁰(^·^OH/H_2_O) ≈ + 2.70 eV and E⁰(^·^OH/OH⁻) ≈ + 1.99 eV at pH = 7. As a result, direct generation of ^·^OH radicals from water by holes in g-C_3_N_4_ is thermodynamically unfavorable. Electrons in the CB of g-C_3_N_4_ react with O_2_ molecules to generate active ^.^O_2_^**−**^ radical species. Since, the CB edge potential of TiO_2_/SiO_2_ (0.03 eV) is higher than potential of E^0^(O_2_/^.^O_2_^**−**^) (-0.33 eV vs. NHE, PH = 7), the electrons in the CB of TiO_2_/SiO_2_ are not suitable to produce ^.^O_2_^**−**^ radicals. Holes in VB of TiO_2_/SiO_2_ react with H_2_O molecules to generate highly reactive ^·^OH. Therefore, the electrons of TiO_2_/SiO_2_ CB with holes of g-C_3_N_4_ VB are recombined. The electrons and holes in CB of g-C_3_N_4_ and VB of TiO_2_/SiO_2_ possess strong potential for generating reactive radical species and facilitating the degradation of environmental pollutants^48^. The Z-scheme mechanism for separation of electrons and holes and charge transfer at heterojunction between TiO_2_/SiO_2_ and g-C_3_N_4_ is proposed.

The Z-scheme charge transfer mechanism in the TiO_2_/SiO_2_/g-C_3_N_4_/MWCNT composite can be described via two possible pathways, as illustrated in Fig. 12. In the first pathway (Fig. 12a), MWCNTs are spatially coupled with either TiO_2_/SiO_2_ or g-C_3_N_4_ and act as a photosensitizer so that charge recombination occurs directly at the heterojunction interface without a mediating step. In the second pathway (Fig. 12b), MWCNT functions as an electron mediator, facilitating the recombination of charge carriers at the TiO_2_/SiO_2_ and g-C_3_N_4_ interface. MWCNT-containing samples exhibited significantly higher visible-light absorption compared to composites without MWCNTs. This enhancement confirms the role of MWCNT as a photosensitizer, promoting activation of the composite under visible-light irradiation. The observed photocatalytic performance is consistent with a direct Z-scheme mechanism^12^. Considering the conductive nature of MWCNT, its E_f_ resides below the CB of g-C_3_N_4_, enabling effective electron transfer from g-C_3_N_4_ to MWCNT. This process suppresses electron–hole recombination by serving as an electron reservoir. Moreover, photogenerated electrons naturally transfer from higher to lower energy states, further supporting the proposed charge migration pathway^12,19,20,31^. Therefore, the photocatalytic activity is enhanced effectively. The electrons react with O_2_ molecules and produce active species radical superoxide for the photodegradation of pollutants^12^.

## Conclusion

A TiO_2_/SiO_2_/g-C_3_N_4_/MWCNT composite was synthesized efficiently through a straightforward hydrothermal approach. The role of MWCNTs was elucidated through optical characterization and photodegradation assessments, revealing their dual function as visible-light photosensitizers and electron reservoirs that improve the spatial separation of photoinduced charge carriers. The optimized composite, with an appropriate MWCNT concentration, demonstrated the highest photocatalytic degradation efficiency of MB, achieving 92% removal within 150 min outperforming the MWCNT-free TiO_2_/SiO_2_/g-C_3_N_4_ composite. Under identical conditions, the photocatalytic degradation of TC in both deionized water and CaCO_3_-containing water also reached 92% with the MWCNT-modified composite. In contrast, the TiO_2_/SiO_2_/g-C_3_N_4_ composite showed lower removal efficiencies of 83% and 75% in pure and hard water, respectively. The superior performance of the MWCNT-integrated system is primarily ascribed to its improved adsorption capacity and charge carrier dynamics, which mitigated the inhibitory effects of ion species in hard water. This indicates that the developed photocatalyst maintains high degradation efficiency even in complex aqueous environments.

## Data Availability

All data generated or analyzed during this study are included in this article and can be available from the corresponding author upon reasonable request.
